# Imperforate anus associated with anomalous pulmonary venous return in scimitar syndrome. Case report from a tertiary hospital in Ethiopia

**DOI:** 10.1186/s12887-019-1643-z

**Published:** 2019-08-27

**Authors:** Tamirat Moges Aklilu, Messele Chanie Adhana, Azmeraw Gissila Aboye

**Affiliations:** 1 Department of Pediatrics and Child Health, Cardiology Unit, College of Health Science, Addis Ababa University (AAU), Addis Ababa, Ethiopia; 2 Department of Pediatrics and Child Health, Residency Program, College of Health Science, AAU, Addis Ababa, Ethiopia; 3 Department of Radiology, College of Health Science, Addis Ababa University, Addis Ababa, Ethiopia

**Keywords:** Scimitar syndrome, Scimitar sign, Anomalous pulmonary venous drainage, Imperforate anus, VACTERL association

## Abstract

**Background:**

Scimitar syndrome is a rare form of partial anomalous pulmonary venous drainage associated with pulmonary hypertension and congestive heart failure that may lead to death in the newborn infant. Although it is described with anomalies of the lung, heart and their vascular structure, extremely rare association with imperforate anus had been reported. The third case of Scimitar syndrome and imperforate anus will be reported in this case report.

**Case presentation:**

A 3 days old male neonate with imperforate anus presented with abdominal distention. Loop colostomy was done to relieve abdominal distension.

The chest x-ray revealed a curved shadow on the right mid lung zone extending to the diaphragm abutting and indenting the inferior vena cava (scimitar sign).

Abdominal ultrasound, transthoracic echocardiography and computerized tomographic angiography confirmed the presence of Scimitar vein and associated dextro-position of the heart, hypoplastic right lung, hypoplastic right pulmonary artery, secundum atrial septal defect with bidirectional shunt, patent ductus arteriosus, pulmonary hypertension, left superior vena cava, and systemic collateral arteries feeding the lower lobe of the right lung.

The rare association of scimitar syndrome with imperforate anus is discussed.

**Conclusion:**

Scimitar syndrome associated with imperforate anus with and without VACTERL association has been reported previously only in four cases. The knowledge of association between imperforate anus and Scimitar syndrome helps for early detection and management of cases. It is recommended to have high index of suspicion in every newborn with imperforate anus to check for symptoms of dextro-position of the heart, right lung hypoplasia which may be indicate scimitar syndrome.

**Electronic supplementary material:**

The online version of this article (10.1186/s12887-019-1643-z) contains supplementary material, which is available to authorized users.

## Background

Scimitar syndrome (SS) is one of the rare variants of partial anomalous pulmonary venous connection associated with anomalies of the lung, heart and their vascular structures in a non-random fashion. The name SS applies to a radiologic appearance of an abnormal right pulmonary vein, in the shape of curved Turkish sword, draining into the inferior vena cava (scimitar sign). It is reported in 3–6% of patients with partial anomalous pulmonary venous connection. The incidence is 1–3 per 100,000 patients [1–3].

During embryogenesis Scimitar vein arises from one or more of the pulmonary veins which drain into superior vena cava (SVC) or inferior vena cave (IVC) instead of joining the common pulmonary vein. The increased venous return may lead to pulmonary volume overload in an already compromised Broncho-vascular structures resulting in a rapid development of pulmonary hypertension. Right lung hypoplasia with dextroposition of the heart, hypoplasia of the right pulmonary artery and right bronchial structures, systemic arterial blood supply to the right lower lung, and various congenital heart disease (ASD, PDA, TOF etc), are among the non-random association [3–5]. SS is a rare form of partial anomalous pulmonary venous drainage and its’ occurrence with imperforate anus with or without VACTERL is extremely rare. To our knowledge only 5 case (2 cases of SS with imperforate anus without VACTERL and 3 cases of SS with imperforate anus in VACTERL) had been reported in the English medical literature [4, 6–9].

Two distinct types of SS had been described in the literature; the infantile and the childhood/adult form. The infantile form is often associated with congenital heart disease and pulmonary hypertension. Patients in this age group are seriously ill with high mortality rate. Patients in the childhood/adult form are asymptomatic in many instances and are diagnosed incidentally [4]. Pulmonary hypertension is a major cause of mortality during infancy [6–8]. The diagnosis of SS should be suspected when the signs of respiratory distress and heart failure present with X-ray findings of dextro-position of the heart and right lung haziness [3].

The aim of this case reports is to draw attention in the medical science towards finding a possible genetic relationship between the common medical condition, imperforate anus with un uncommon one, Scimitar syndrome.

## Case presentation

A 3 days old male neonate from southern Ethiopia presented to a nearby health institution with difficulty in sucking, failure to pass meconium and abdominal distension since birth.

Physical examination revealed imperforate anus. After referral to a tertiary hospital, loop colostomy was done to relieve respiratory distress. Ampicillin and Gentamicin were started because of persistent respiratory distress and a possible association of sepsis. After four days of treatment, the antibiotics were changed to ceftriaxone and metronidazole because of colostomy site infection. However, after completing 8 days of antibiotic treatment the respiratory distress had not resolved. Treatment for sepsis was continued.

The baby was born at term at a government health institution (health center) to a 35 years old para VII mother through spontaneous vaginal delivery. The birth weight and APGAR score were not recorded. The mother’s antenatal care and follow up were done at a local health center where Tetanus Toxoid vaccine was given. Contraceptive injection (Depo-Provera) was given for three years but was discontinued six months prior to the current pregnancy. There was no maternal history of alcohol consumption, smoking cigarette, diabetes mellitus, hypertension or human immunodeficiency virus infection.

There was no history of consanguinity and no family history of cardiac disease. The patient was exclusively breast fed since birth. Physical examination on admission: -Vital signs: - Temp- 37.2^o^c, RR-68/m, AHR-160 bpm, BP-74/46 mmHg, SPo2–92%, Weight- 3.8 kg (50th–75th centile), Length-52 cm (50th–75th centile), Head circumference-36 cm (50th–75th centile).

There were no gross dysmorphic features. The lympho-glandular system was normal. Inter-costal retractions and relative dullness with reduced air entry on the right-side lung field were noted. Peripheral pulses with strong volume were palpable in all accessible area. The precordium was quiet. The point of maximal impulse was felt at the 4th intercostal space medial to mid clavicular line. There was no thrill detected. The heart sounds were normal and without any murmur or gallop on auscultation. There was no pedal edema and no hepatosplenomegaly.

**Fig. 1:**
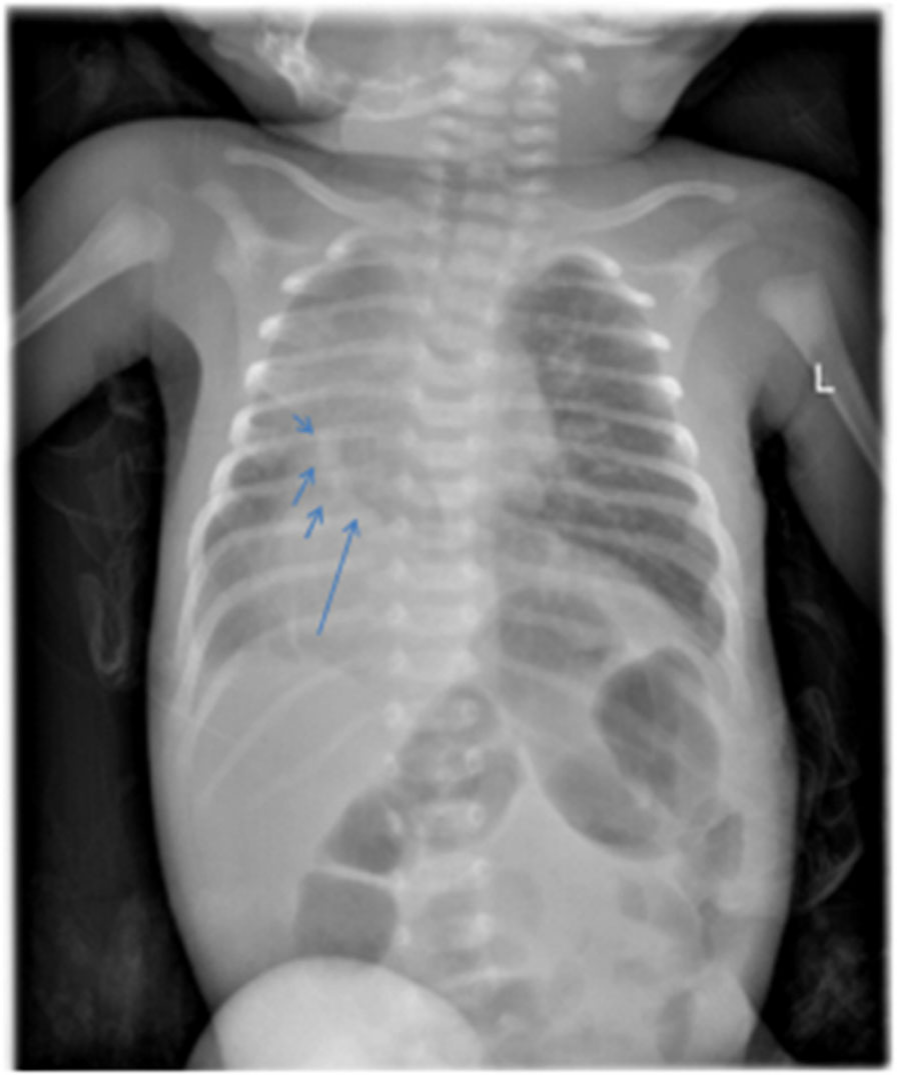
Chest X-ray imaging showing “Scimitar sign” in chest X-ray. in the right mid lung zone through the lower lung region of the right cardio phrenic angle

External genital examination showed normal male phenotype. There was low type imperforate anus. No other gross dysmorphisms were detected on physical examination. Investigations:

Hemoglobin and C-reactive protein ranged between 12.7–15.2 g/d and 12-24 mg/dl respectively. WBC-6700/mm^3^, platelet-203,000/mm^3^, MCV-89.2, Serum creatinin-0.5 mg/dl. Abdominal ultrasound performed using 5 MHz probe (Sono-scape ultrasound) showed minimal inter-loop fluid collection. Chest x-ray showed abnormal curvilinear radiographic shadow in the right mid lung zone extending across the diaphragm in to the inferior vena cava (Figure 1). Echocardiographic examination was performed by an experienced pediatric echocardiographer using Philips Ultrasound 22,100 Bothell USA MOD iE33 system with a 2.5-mHz transthoracic probe. Apical and subcostal four-chamber sections were obtained. Two-dimensional(2D) transthoracic echocardiography were used to assess the characteristics of the atrial septal defect, pulmonary vein connections, inferior vena cava drainage, presence of obstruction in the venous collector, and dilation of the right cardiac chambers. The diameters of the main and branch pulmonary arteries were determined in the parasternal view. Right pulmonary arterial hypoplasia was defined as a diameter < 5 mm (MPA), < 3.5 mm (RPA) at birth [10, 11].

Pulmonary hypertension was suspected when the trans-tricuspid regurgitation velocity was > 3.4 m/s by Doppler method. Using continuous wave and color doppler imaging in an apical four chamber view, the sample volume was placed at the height of the tricuspid valve and the pressure gradient between the right atrium and the right ventricle was determined using Bernoulli’s method. The result was multiplied by a correction factor of 1.23 to obtain the pulmonary artery systolic pressure [1].

**Fig. 2:**
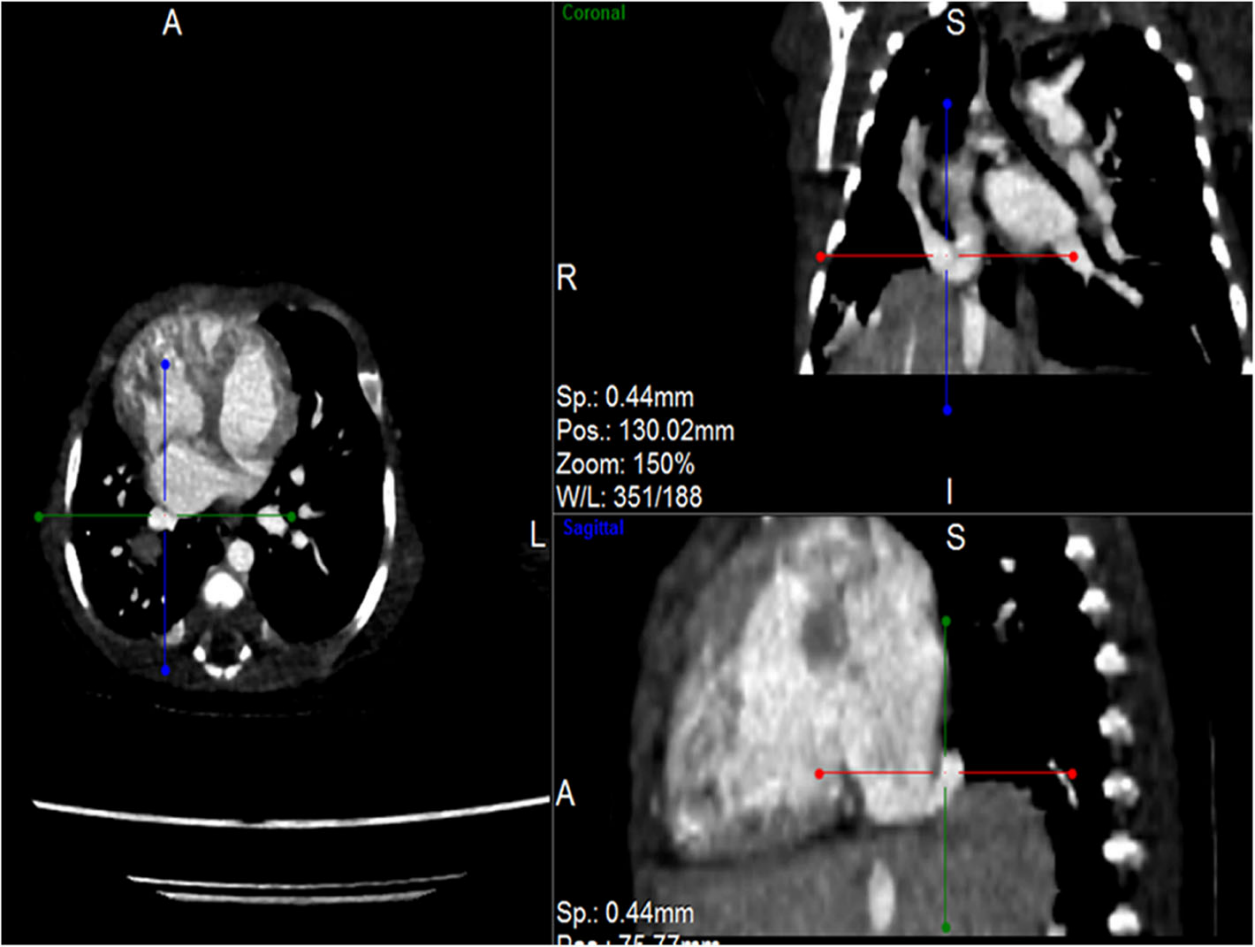
CT angio-axial, coronal and sagittal images demonstrating the scimitar vein

**Fig. 3:**
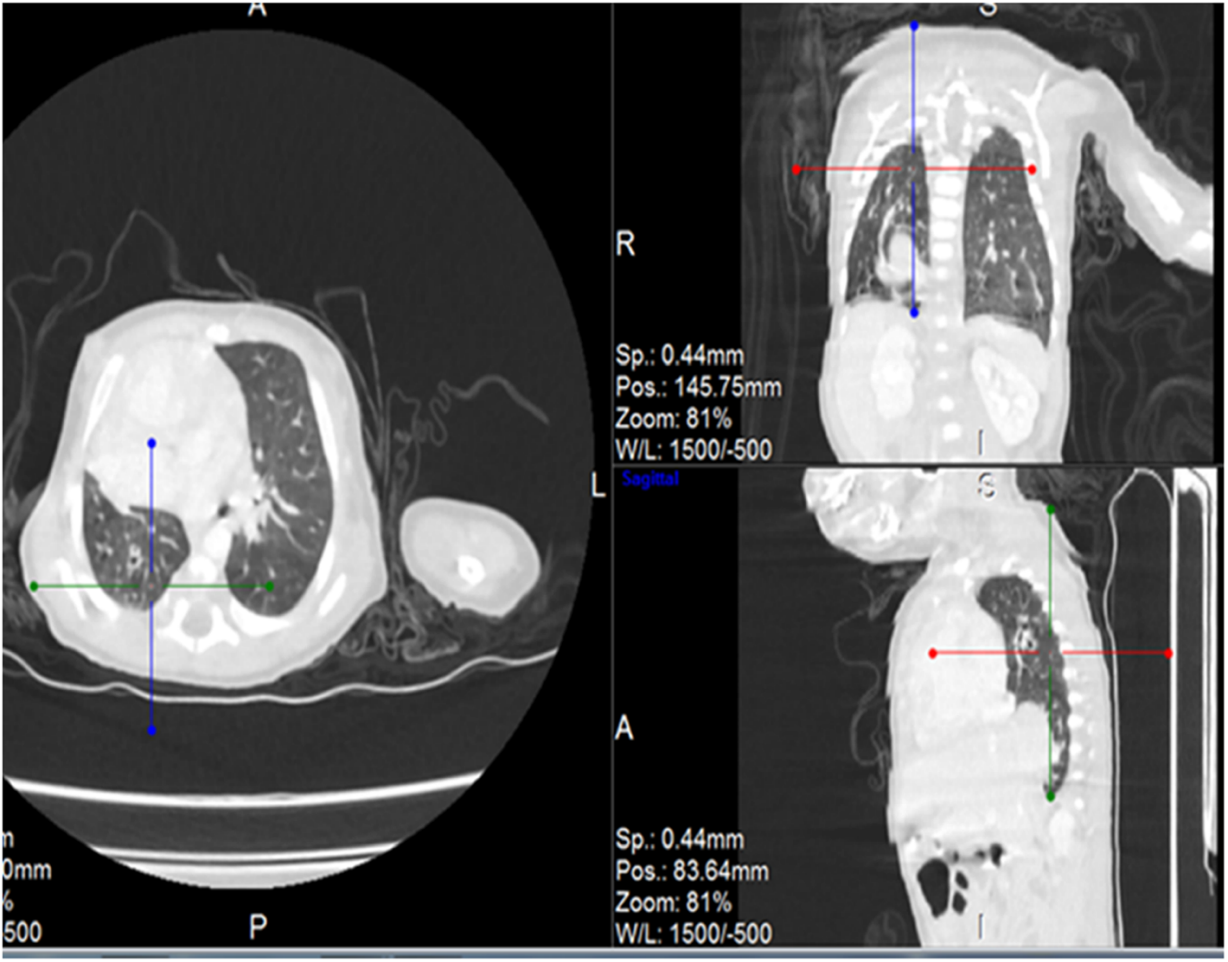
CT angio-axial, coronal and sagittal images demonstrating hypoplastic right lung

**Fig. 4:**
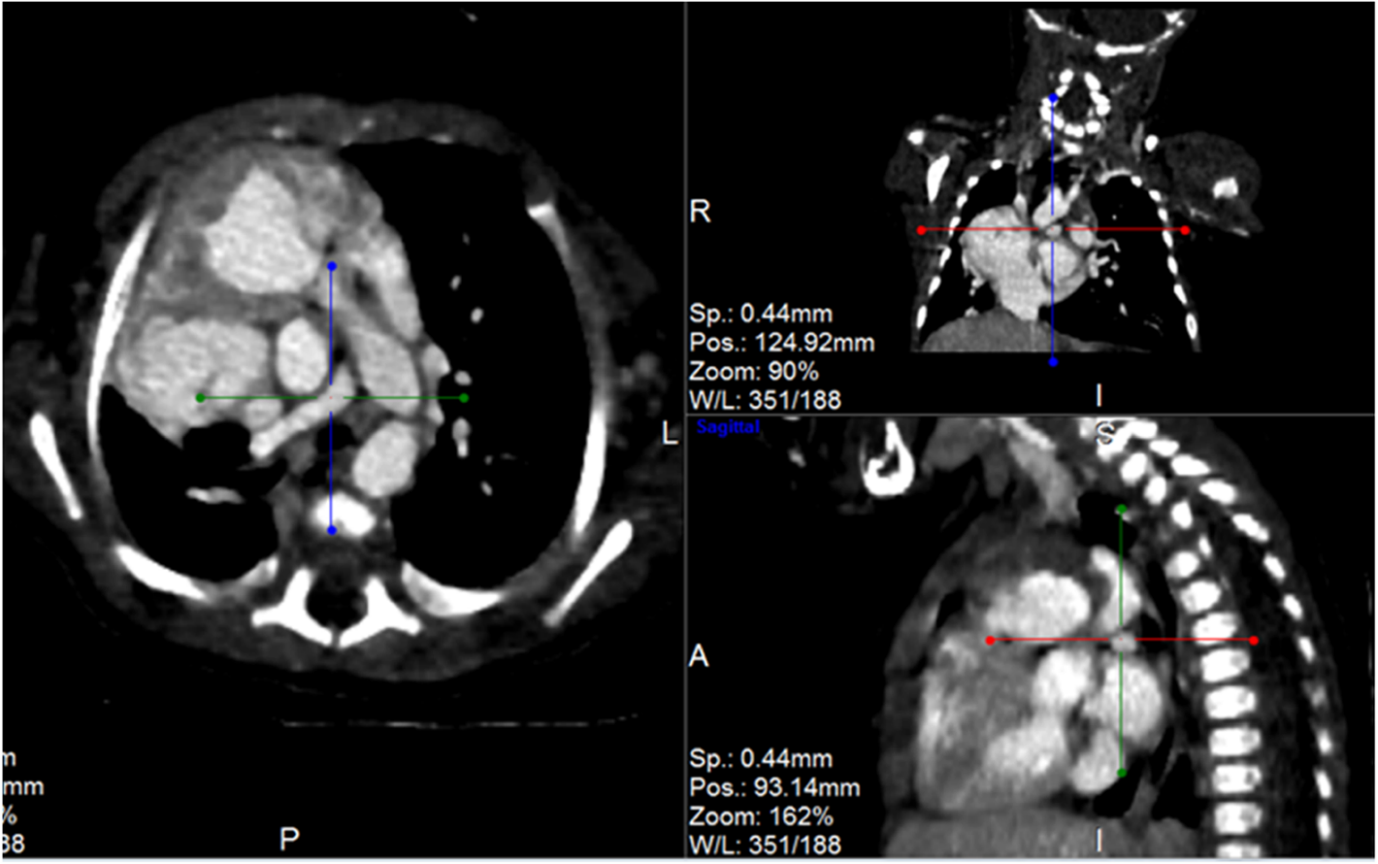
CT angio-axial, coronal and sagittal images demonstrating markedly hypoplastic RPA

**Fig. 5:**
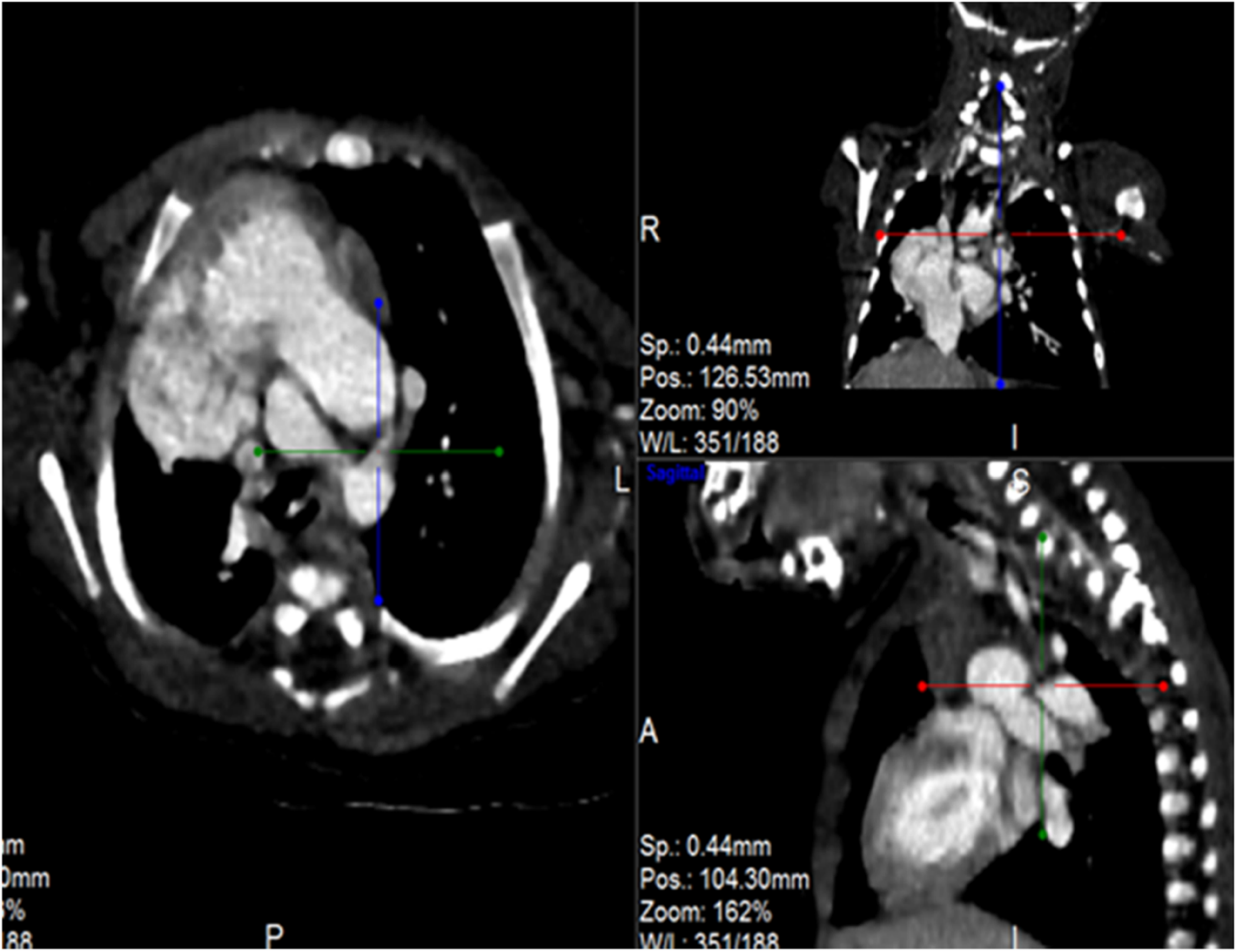
CT angio-axial, coronal and sagittal images demonstrating PDA

**Fig. 6:**
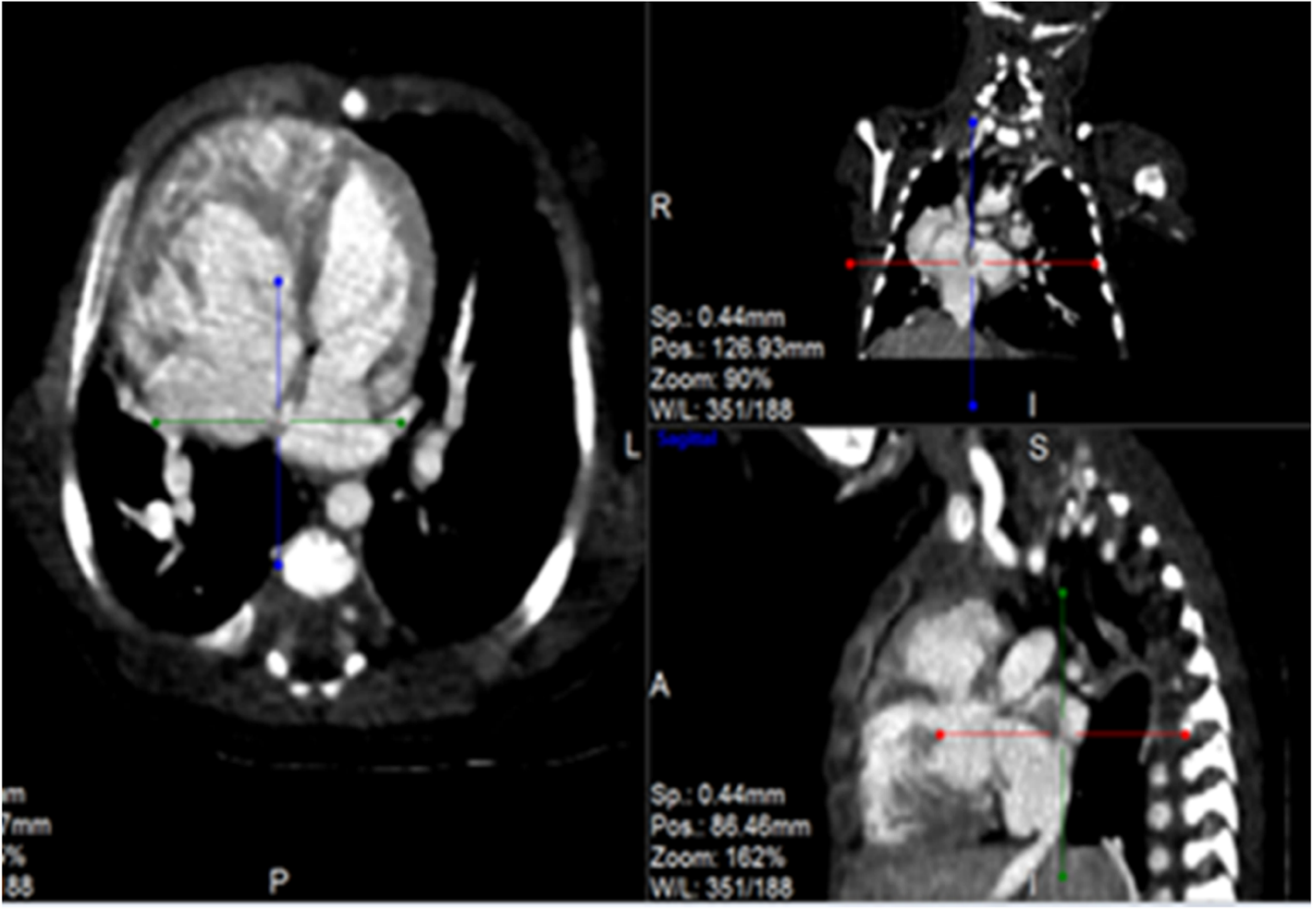
CT angio showing small secundum ASD

**Fig. 7:**
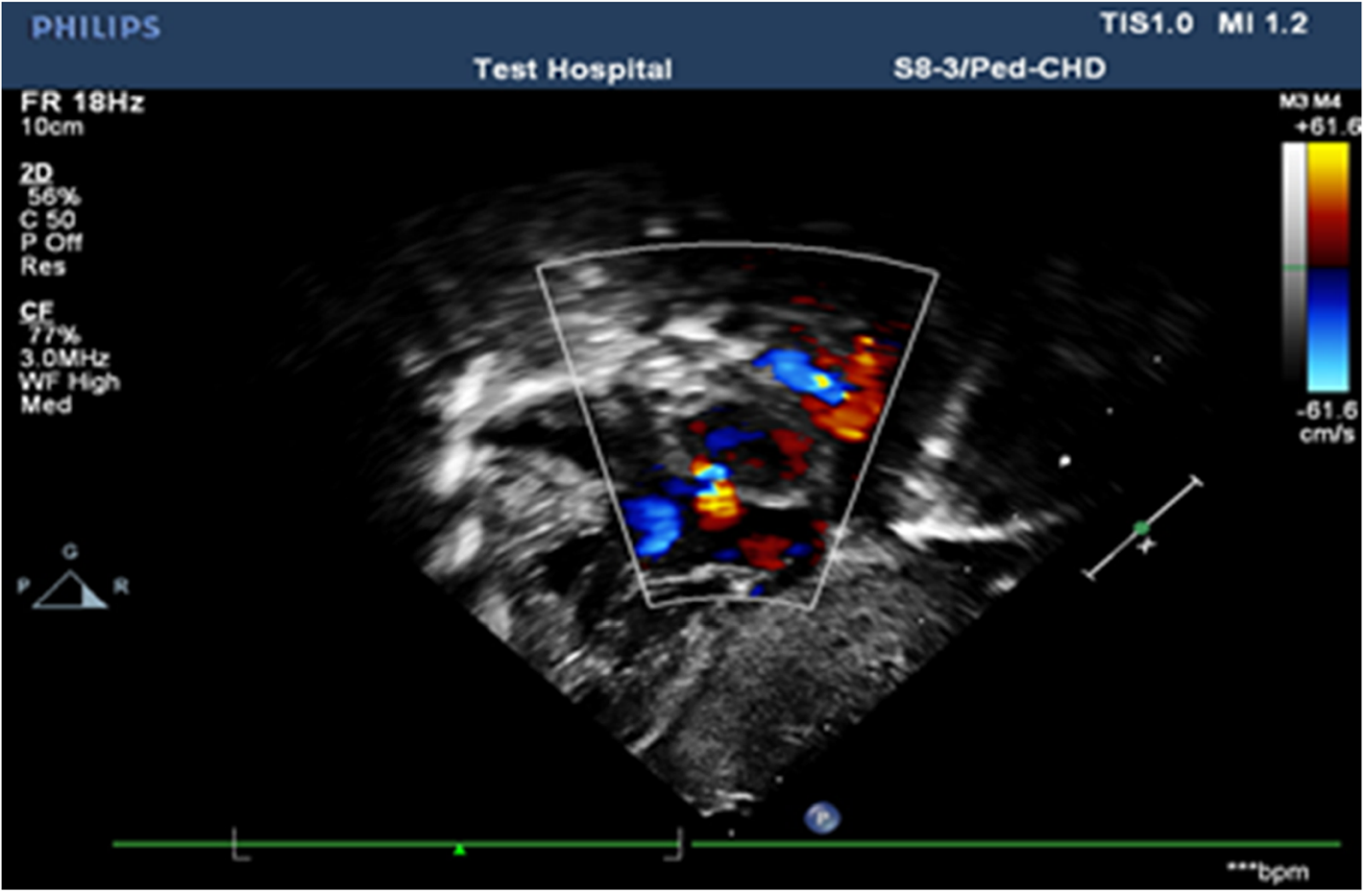
2D echocardiographic image showing small 2^o^ ASD

**Fig. 8:**
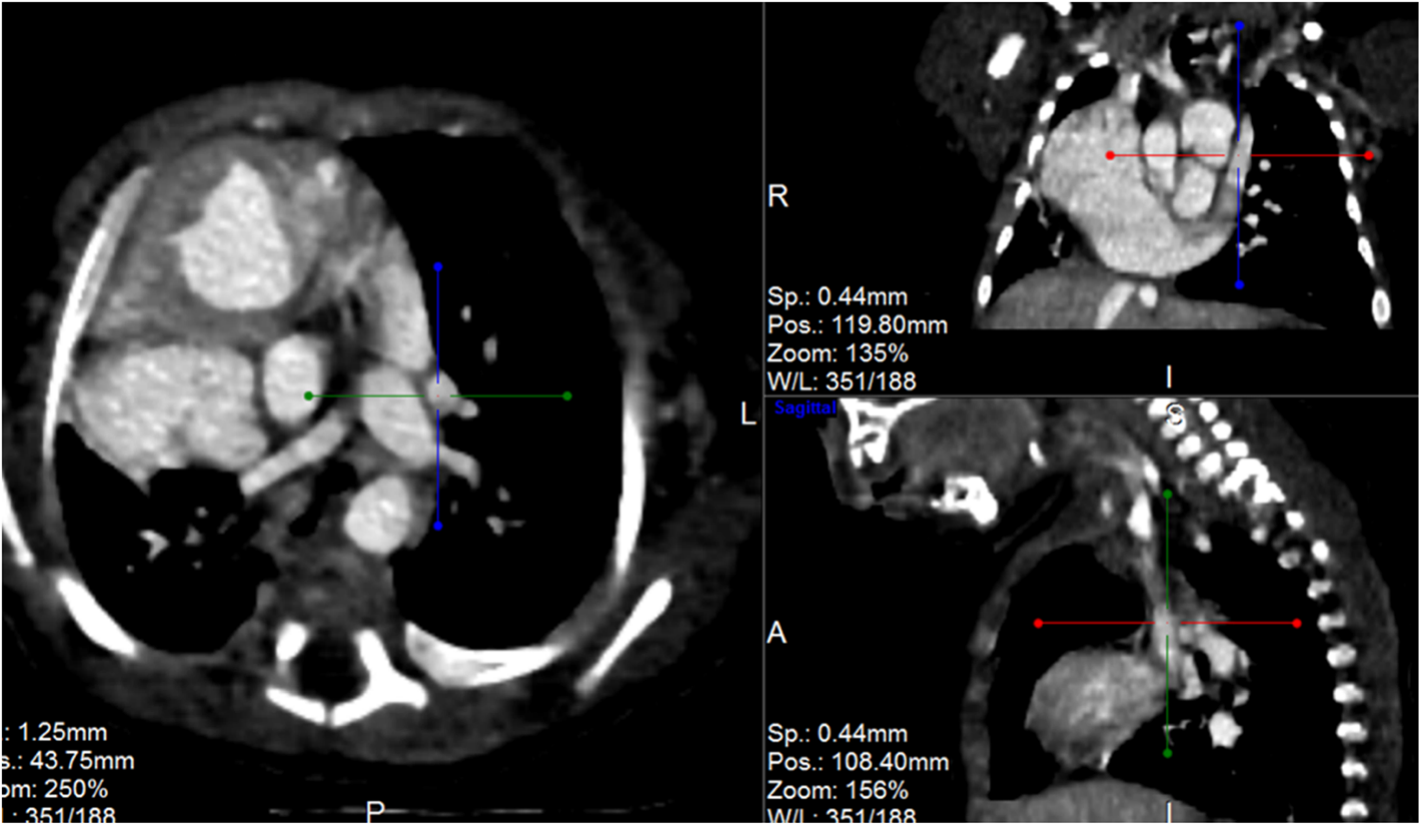
CT angio-axial, coronal and sagittal images demonstrating left SVC and dilated coronary sinus

**Fig. 9:**
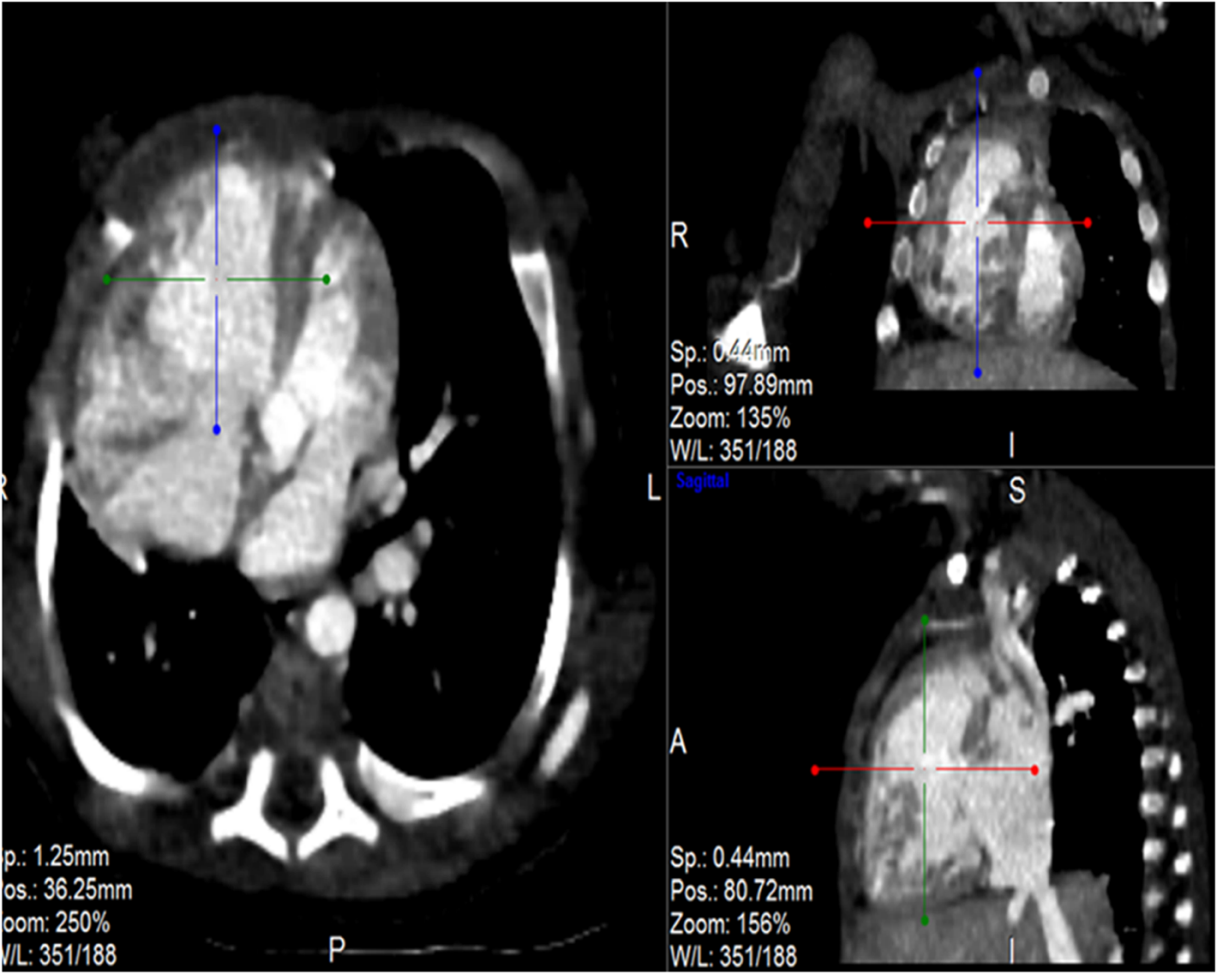
CT angio-axial, coronal and sagittal images demonstrating dilated right side chambers

**Fig. 10:**
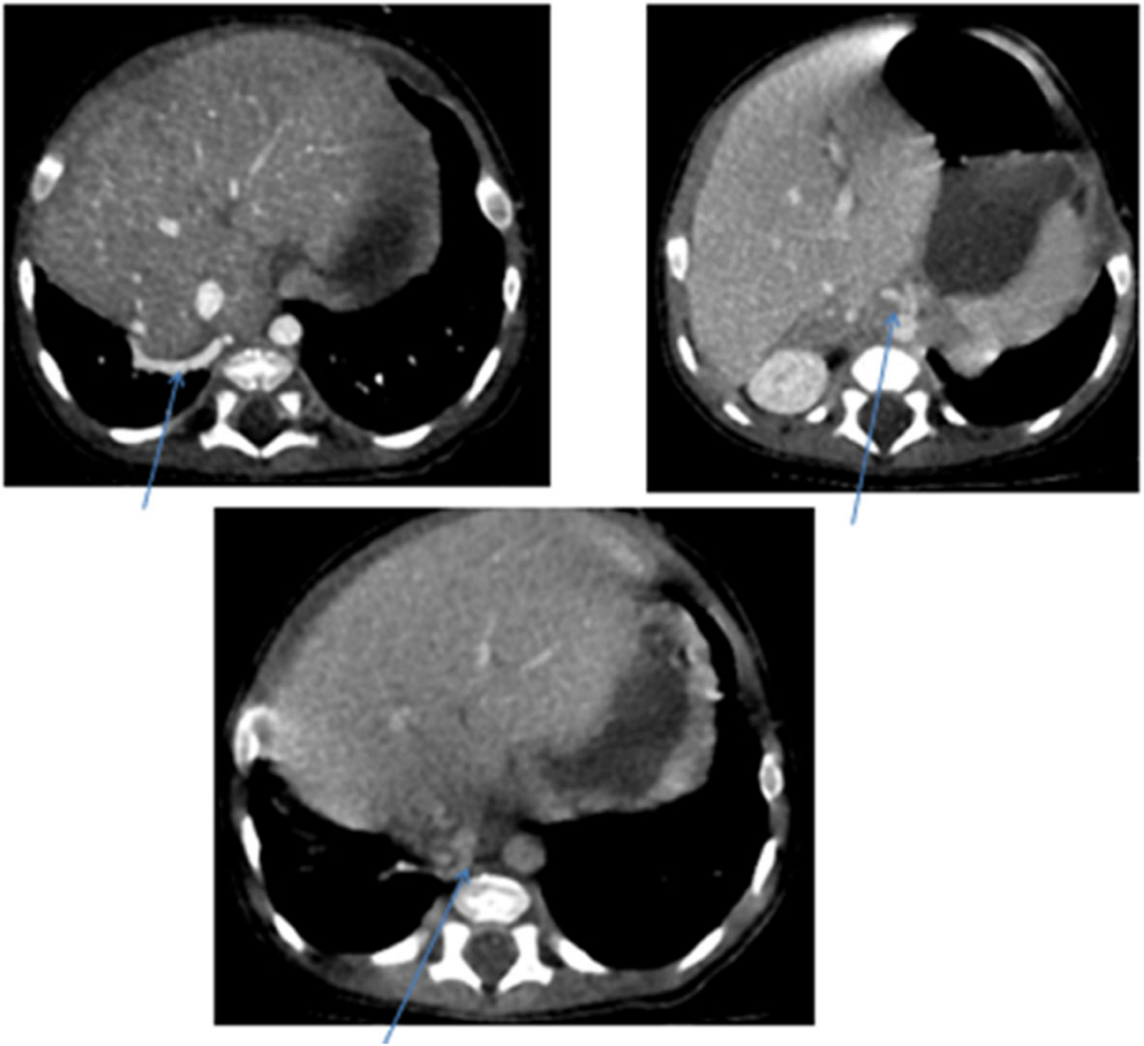
CT-angio showing systemic collateral arteries feeding the right lower lung arising from infra-diaphragmatic aorta


Post contrast computerized tomography of the chest (CT angiography) was performed using a 64 detector row scanner (GE medical systems; Optima CT 660).The scan was acquired in helical mode and was performed using pediatric protocol with parameters of KV 120 and MA 130.The thorax from the thoracic inlet to the upper abdomen was included. CT scan was done to confirm the findings found in the other imaging modalities and to look for additional associated findings. CT scan showed Scimitar vein arising from right middle lung zone draining the whole right lung as a single right pulmonary vein into the IVC at the level of its junction with the right atrium (Fig. 2 and Additional file 1). The right lung looked relatively small compared to the left and there was secondary dextro-position of the heart with right side mediastinal shift (Fig. 3). The RPA looked markedly hypoplastic (Fig. 4). There was small PDA (Fig. 5) and secundum ASD (Figs. 6 and 7). CT angio also showed left superior vena cava draining into the coronary sinus which is markedly dilated (Fig. 8). The right heart chambers looked markedly dilated with right ventricular hypertrophy (Fig. 9). Systemic collateral arteries arising from abdominal aorta drained into the right lower lung (Fig. 10). Course in the hospital: after confirmation of the diagnosis of pulmonary hypertension, intravenous furosemide 1 mg/kg/dose BID was started. Sildenafil 0.5 mg/kg TID was also initiated orally. The respiratory distress resolved (RR ranging between 72/m and 48/m). The temperature dropped between 35.8o^c^ and 36.7o^c^. Oxygen saturation ranged between 92 and 99%. Patient was discharged to be followed in the cardio-thoracic section for possible surgical intervention. Also referred to general pediatric surgery for correction of imperforate anus.

**Additional file 1:** Video showing scimitar vein draing in to upper part of IVC. (AVI 7284 kb)


## Discussion and conclusion

The neonate was first brought to a health care facility because of difficulty in sucking, failure to pass meconium and abdominal distension which later found to be due to imperforate anus. The partial anomalous pulmonary venous drainage arising from the right mid lung to the IVC (Scimitar sign), dextro-position of the heart, hypoplastic right lung, hypoplastic right pulmonary artery, PDA, Secundum ASD, dilated right cardiac chambers, left superior vena cava, systemic-collateral arteries feeding the right lower lung from the infra-diaphragmatic aorta found in our patient, are all described in scimitar syndrome.

In this case report the authors picked un uncommon association of a rare clinical condition (SS) with relatively common one (imperforate anus). The major limitation of the current case report is that, it lacks genetic testing that may show an association between SS and VACTERL. Therefore, further studies are needed to better understand if SS and VACTERL share common pathogenetics. Sildenafil dose is not monitored using serum level of this drug; therefore, we do not know whether its pharmacologic efficacy is achieved or not.

The different anomalies of SS were described with different frequencies. Mathew J et al. reported hypoplasia of right pulmonary artery in 60% of cases on angiography. Associated ASD was reported in 40% of patients with SS [1]. Others reported dextrocardia or mesocardia in 70%, and atrial septal defect in 70% [12]. Systemic collateral artery arising from infra-diaphragmatic aorta feeding the right lower lung fields were reported in 48% of cases.

Left side SVC found in our patient was also reported by Mathew J et al [1]. Associated imperforate anus was also previously reported. Rezaei M et al. reported a male neonate with imperforate anus and SS at birth. The age of diagnosis, gender and clinical presentations, were similar to the case presented in this report. Imaging modalities revealed right lung hypoplasia, absence of right pulmonary artery, and drainage of the small remnant of the right lung directly into the inferior vena cava. Opacity of the right hemi thorax on radiograph is due to right mediastinal shift and sequestration of the right lower lobe. The pattern of congenital heart disease was different in the case reported here. Coarctation of the aorta, right pulmonary artery stenosis, and right to left ductal flow previously reported were not detected in our patient [6]**.** The second case of scimitar syndrome and imperforate anus were reported from South Korea. Since the report was in Korean language, clinical details are not included in this report [4].

Christian J et al. reported a neonate with probable prenatal diagnosis of VACTERL association and small left-sided cardiac structures, which on postnatal angiography were found to be part of a SS. They recommended that SS be considered in the prenatal and post-natal evaluation of VACTERL association [7]. Puneet A et al. described a case of SS with imperforate anus with VACTERL association. The imperforate anus was detected and managed early. However, the anomalous pulmonary venous return was diagnosed at the age of 4 years. The patient reported also had another congenital heart disease (Tetralogy of Fallot) and large ASD shunting bi-directionally), dextro-position of the heart, hypoplastic right lung with SS, hypoplastic right pulmonary artery, large aorto-pulmonary collaterals from the abdominal aorta supplying the entire right lung [8]. Though imperforate anus was detected at birth the diagnosis of SS was delayed. Punnet A et al. suggested that SS is one of the six component features of VACTERL association and yet this association might have been overlooked. Our patients had no full description of VACTERL association except imperforate anus and congenital heart disease. Whether SS and VACTERL association share a common pathogenetic pathway or one is the feature of another as suggested by Puneet A et al. remains unanswered. The findings in this case report are similar to those found in previous reports suggesting a probable non-random association between SS and imperforate anus. The authors recommend to have high index of suspicion in every newborn with imperforate anus to check for sign and symptoms of dextro-position of the heart, and right lung hypoplasia which may be indicate scimitar syndrome. Further studies are also needed with supplemental genetic testing to see if there is a common genetic pathway for the two conditions.

## Data Availability

The data set supporting the conclusions of this article are included within the article.

## Abbreviations

ANC: Antenatal care
APGAR score: Activity, Pulse, Grimace, Appearance, Respiration
ASD: Atrial septal defect
BID: (bis in dei), twice daily
CT: computerized tomography
CXR: Chest X-ray
HIV: Human immunodeficiency virus
IV: Intravenously
IVC: Inferior vena cava
MCV: Mean corpuscular volume
MPA: Main pulmonary artery
PDA: Patent ductus arteriosus,
RPA: Right pulmonary artery
SPo2: Saturation of peripheral oxygen
SS: Scimitar syndrome
SVC: Superior vena cava
SVD: Spontaneous vaginal delivery
TASH: Tikur anbessa specialized hospital
TID: (ter in dei), Three times daily
TOF: Tetralogy of Fallot
TT: Tetanus toxoid
VACTERL association: Vertebral abnormalities, anal atresia, cardiac (heart) defects, tracheal-esophageal abnormalities, renal and radial abnormalities, limb abnormalities
WBC: White blood cell count
